# Nurse rostering: understanding the current shift work scheduling processes, benefits, limitations, and potential fatigue risks

**DOI:** 10.1186/s12912-024-01949-2

**Published:** 2024-04-29

**Authors:** Lauren A. Booker, Jane Mills, Melanie Bish, Jo Spong, Melissa Deacon-Crouch, Timothy C. Skinner

**Affiliations:** 1 School of Psychology & Public Health, La Trobe University, Bendigo, VIC 3552 Australia; 2 Department of Rural Health Sciences, La Trobe Rural Health School, La Trobe University, Bendigo, VIC 3552 Australia; 3 Department of Psychology, Centre for Health and Society, University of Copenhagen, Copenhagen, Denmark; 4 Institute for Breathing and Sleep, Austin Health, Melbourne, 3084 Australia

**Keywords:** Sleep, Healthcare workforce, Nurses, Shift work, Fatigue, Occupational health and safety

## Abstract

**Background:**

Implementing appropriate shift work schedules can help mitigate the risk of sleep impairment and reduce fatigue of healthcare workers, reducing occupational health and safety risks. In Australia, the organisation has a responsibility to make sure all reasonable measures are taken to reduce fatigue of staff. Therefore, it is important to assess what the current rostering processes is for staff responsible for creating the rosters for nurses.

**Aim:**

The aims of the project were to understand (1) who creates the rosters and what the process is, (2) what training and knowledge these staff have in establishing rostering schedules that optimise the sleep and wellbeing of staff, and (3) what the benefits and limitations are of current rostering practices.

**Methods:**

Findings were generated through semi-structured interviews, using cluster coding to form categories. Twenty four nurses responsible for rostering staff were interviewed from three different sites in Victoria (one metropolitan and two regional/rural hospitals). Data was analysed using selected grounded theory methods with thematic analysis.

**Results:**

The common themes that came out of the interviews were that rostering staff were under prepared, unaware of fatigue and safety guidelines and polices from governing bodies and had not received any education or training before taking on the role. The most common rostering style was self-rostering, where staff could submit preferences. However, there were concerns about staff fatigue but were divided as to who should be responsible, with many saying it was up to staff to preference shifts that they could cope with. The final theme was cultural barriers to change.

**Conclusion:**

While self-rostering resulted in staff having more freedom and flexibility,  shift preferences may be influenced more so by a need to fit with lifestyle rather than to minimise fatigue and increase safety in the workplace. Greater consideration of the impact of shift work schedules on fatigue is required to ensure that the layers of clinical governance in health care organisations minimise the risk of occupation health and safety issues for employees delivering direct patient care.

## Introduction

Shift workers are at greater risk of occupational hazards such as workplace injury, absenteeism, workplace errors as well as motor vehicle accidents [1–3]. While there is a diverse range of negative impacts from working shift work, the consequences of impaired sleep can affect the physical and mental health of workers, contributing to fatigue and mental exhaustion, cognitive performance, and overall quality of life [4, 5]. In addition, impaired sleep contributes substantially to loss of productivity, absenteeism, and workplace-related accidents [6]. Fatigue can occur when there is prolonged sleep loss and/or disruption of the internal body clock as well as mental or physical activity, leading to a state of exhaustion that reduces a person’s ability to perform work safely and effectively [7]. Research shows that staff who are fatigued and sleep impaired are at considerably higher risk of making a medical error [8]. Further, workers with sleep problems have a 1.62 times higher risk of being injured at work and approximately 13% of work injuries can be attributed to sleep impairment, compared to workers without sleep problems [9].

Occupational health and safety organisations, such as SafeWork Australia and WorkSafe Victoria, provide comprehensive guidelines on fatigue management, which outline the organisational, work, and personal factors that can lead to fatigue [10, 11]. The Victorian Occupational Health and Safety (OH&S) Act 2004 and Occupational Health and Safety Regulations 2017 [12, 13], provides employers with a legal framework to ensure that reasonable measures are taken to ensure safe working conditions and injury prevention in the workplace by providing the information, instruction, training, and supervision necessary to enable employees to work in a way that is safe and without risks to health including managing fatigue risk. This includes how to identify the causes of fatigue and the potential consequences; the application of relevant legislation; and the development and implementation of risk management strategies. Such strategies may focus on shift work scheduling to eliminate or minimize fatigue-related risk so far as reasonably practicable. Employees are also obligated to take all reasonable steps to take care of their own health and safety and that of anyone else that may be affected by their actions in the workplace. Implementing appropriate shift work schedules can help mitigate the risk of sleep impairment and reduce fatigue [14, 15]. For example, rotating between day and evening shifts can be worse for cognitive performance than working night shifts [16, 17]. Therefore, having shifts blocked together and forward rotating (from morning to evening and then to nights) can alleviate some of the circadian disruption workers may experience [15].

For these reasons, it is important to assess what the current processes are for roster creation of nurses, whether there is knowledge and/or training of staff on how to create safe shift work schedules and, if there is scope to improve these practices to reduce fatigue and its consequences. Currently however no research has documented and reported this. Thus, the aims of this project were to understand (1) who creates the rosters and what the process is, (2) what training and knowledge these staff have in establishing rostering schedules that optimise the sleep and wellbeing of staff, and 3)the benefits and limitations are of current rostering practices.

## Materials and methods

### Study design and participants

A qualitative design approach, using an instrumental single case study design was used [18]. This was chosen as it provides an in-depth descriptive understanding from multiple perspectives, who are bound by a shared phenomenon. These are then grouped into a single case study such as by occupation, person or environment [19, 20]. In this study the case was made up of three different sites in Victoria, Australia (one metropolitan and two regional/rural hospitals). These sites were targeted for recruitment to reflect diverse working contexts and to form the group, across medical, surgical and specialty services. The metropolitan hospital size was approximately 900 beds, treating around 90,000 patients, one of the regional hospitals had 700 beds treating around 45,00 patients and the second rural hospital had 200 beds, treating around 4,700 patients. Participants had to be currently working at one of the participating sites and be responsible for creating the shift work rosters for nurses on their department/ward, at one of the targeted sites, in Victoria Australia. There were no other inclusion criteria.

### Data collection

Participating healthcare sites assisted in the identification of staff who were responsible for creating the roster on each department/ward. To ensure that the project remained impartial from the participants’ employer, sites did not recruit or collect data directly but instead circulated an expression of interest email to those staff identified which invited them to participate in the study. Those staff that wished to participate then expressed interest directly to the research team via email. Potential participants were then given information outlining what the study entailed and supplied with a copy of the Participant Information Consent Form to review and sign. Once consent had been obtained, an appropriate day/time was scheduled to conduct the interviews. The interview questions were created by the research team to reflect the information required to meet the aims. These were semi-structured questions that covered topics including how staff were allocated the role of creating the rosters on their ward, what education or guidance was received, what the process was and rules they followed to create the rosters (including shift patterns, skill-set etc.), if they had knowledge of legislation and fatigue management policies in regards to safe shift work schedules, if they were concerned about fatigue of staff, or if there were any improvements or barriers to improve the process. An interview script was developed, with appropriate prompts, to ensure that all interviews followed a similar structure and that all the interview questions were covered. Interviews were conducted by a single person to ensure consistency. Interviews took around 30 min to complete and were conducted remotely via phone/ videoconferencing (such as Zoom) and recorded for transcribing verbatim later. Data collected was then de-identified and a unique ID number applied. Participation in the study was voluntary. Interviews were conducted between February and June 2023.

### Data analysis

Interviews were recorded and transcribed verbatim. Selected grounded theory methods, using thematic analysis was used to analyse the transcripts of the semi-structured interviews to generate common concepts and patterns within the data [21, 22]. Grounded theory is an inductive approach that allows for the development of theories, instead of testing an existing one. This method of analysis included identifying patterns, and clustering to form categories. A preliminary round of coding and categorization was conducted by the team to identify initial themes and then the clustering of codes to form categories. Data analysis was conducted by two authors independently to promote consistency and dependability. The final themes and subthemes identified were assessed and verified by the members of the research team.

### Rigour

This study strengthened its trustworthiness and rigour by ensuring credibility, transferability, confirmability, and dependability [23]. Credibility was enhanced from the triangulation of the data collected from a purposive sample recruiting participants with the best imaginable knowledge of the research question was deemed suitable as well as ensure variation and experience at different hospitals. Generalisability was improved by using an instrumental single case study, which was chosen as an appropriate design because the aim of the study was to move beyond the case itself to develop findings that are transferable to other sites and other disciplines in the Victorian healthcare system [24]. Data generated from each of the sites in the group were compared to each other to identify similarities and differences within the single case of rostering practices for shift working nurses in Victoria. Dependability was achieved by following a logical and semi-structured interview process for all participants and confirmability was ensured by reporting the interviews verbatim and themes drawn directly from the data, thus ensuring the research team are not interpreting them.

### Ethical considerations

This study was conducted in accordance with the Declaration of Helsinki. We confirm that all methods were performed in accordance with the relevant guidelines and regulations. Ethical approval was granted by the La Trobe University Human Research Ethics Committee (Ethics number: LNR/90,536/BH-2022-341572). The participants of the study provided written informed consent, who were informed that participation was completely voluntary, and that they could withdraw at any time during the process until publication without reason. The participants were guaranteed confidentiality, and that participation was not related to or would interfere with their relationship with their employer.

## Results

A total of 24 interviews were conducted, with participants representing a variety of practice contexts including Intensive Care Unit (ICU), Emergency Department, operating theatre, and aged care. The common themes that came out of the interviews were that rostering staff were under prepared and lacked training from their employer, staff self-preferencing their rosters was common, there were concerns about staff fatigue, but that staff fatigue responsibility was divided and that there were cultural barriers to wanting change.

### Appointment of rostering staff

There was a variation as to how staff were allocated the responsibility of creating the rosters for their ward/department including it being part of their management role or because they were back filling the role when a more senior staff member was on leave and ended up staying in the role. For example,*” The role is often dreaded by others as it is not exciting and have to put up with complaints from staff.” (Participant 17, female, Associate Nurse Unit Manager, Specialty ward).* In addition, a smaller number of participants also volunteered for the position because either no one else wanted to do it or because they enjoyed a challenge, with one participant stating, *“[I] enjoy puzzles and organising things.” (Participant 9, female, Associate Nurse Unit Manager, Perioperative ward ).* Almost all participants said that doing the rosters was a part of their workload and that they were given approximately a day month to complete the task. Some however, said that this was not enough time and that they had to do it at home. *“It takes at least 3 days a month- sometimes I have to do them at home or on my days off to fit it in because the hospital has a set date that rosters need to be in by.” (Participant 22, female, Associate Nurse Unit Manager, Speciality ward).*

### Under preparedness of rostering staff

When participants took up the role, all participants stated that they received no training or guidance on how to create safe shift work schedules from their employer. Instead, participants received an internal hand-over from a previous staff member, such as information on staff preferences and shadowing them while they completed their first roster. When asked what sort of training they wish they had received, the vast majority stated that they couldn’t think of anything that would have been beneficial because was each ward is different. For example, *“I don’t know what training you could give as each ward is different.” (Participant 20, female, Registered Nurse, ICU).* Instead participants stated that it was more about getting to know staff preferences and requests than acquiring technical or evidence-based skills. For instance, *“It’s about knowing staffs’ likes and dislikes and arrangements that are in place.” (Participant 24, female, Associate Nurse Unit Manager, Speciality ward).* However, a couple mentioned that training on software programs such as KRONOS, finance, and budgeting, managing staff preferences and how to manage lard wards would have been beneficial. *“All new staff should learn how to build and manage large wards and staff preferences. While also managing the budget and costs i.e. ADO’s need to be taken into consideration as well as annual leave, if staff don’t take it themselves, you need to roster it on.” (Participant 23, female, Nurse Unit Manager, Surgery ward).*

When it came to knowledge of fatigue management policies, most participants assumed that they existed in their organisation but were not aware of them. Two people stated that they had read a policy but that it was out of date or covered general rosters guidelines, not fatigue related factors, such as how to use Kronos (shift rostering software program). A few mentioned that the Australian Nursing and Midwifery Federation (AMNF) had guidelines and that their Employment Bargaining Agreement (EBA) agreement had conditions around shift work schedules that they followed. However, it still come down to staff preferences, which overruled these, with one participant saying, *“I try to adhere to rules such as early shift before a day off and 2 days off in a row after a night shift unless staff preference otherwise.” (Participant 6, female, Associate Nurse Unit Manager, Acute nursing unit)*.

### Roster allocation

The most common rostering style was self-rostering, where staff had the ability to request which shifts they preferred over that rostering period. The percentage of requests granted ranged from 50% to almost all shifts (where possible) would be approved, *“Staff can self-allocate 50% of their EFT themselves.” (Participant 5, female, Associate Nurse Unit Manager, Speciality ward).* And many thought that this style of rostering allowed for a better work/life balance and gave the staff more control. As one participant said *“Self-rostering makes staff feel empowered. It’s been around for about 12 months. They don’t feel like the rosters are against them, limits room to complain. So, it makes the process transparent so everyone can see each other’s requests are and what has been given.” (Participant 17, female, Associate Nurse Unit Manager, Specialty ward).*

Others thought they had no option because if staff didn’t get their requests, they would just call in sick. *“Try to give all staff requests or they will not turn up anyway.” (Participant 10 female, Nurse unit manager, Speciality ward).*

Another important driver for some were that by granting shift preferences it would help with staff retention with multiple participants stating, *“It’s all about recruitment and retention of staff. It is very competitive, and you need to be appealing to attract workers.” (Participant 6, female, Associate Nurse Unit Manager, Acute nursing unit)* or thinking *“It is nice to give staff the ability to dictate, makes for a happy environment and helps with staff retention.” (Participant 16, female, Associate Nurse Unit Manager, Medical ward).*

But some participants were more strict in regards to approving requests because the hospital needs shifts covered, *“Staff have to do it - night shift is part of their job.” (Participant 24, female, Associate Nurse Unit Manager, Speciality ward)* and another stating *“At the end of the day the hospital has to run, it is still a business.” (Participant 9, female, Associate Nurse Unit Manager, Perioperative ward).*

### Concerns of staff fatigue

When it came to fatigue concerns, participant responses were divided. Some were not concerned by staff fatigue due to their shift work schedules saying, *“No, I don’t think of fatigue too much, it’s not a priority.” (Participant 22, female, Associate Nurse Unit Manager, Speciality ward)*, or *“No, we safeguard this by having the right mix of skills and staff breaks and grads only do 0.8 EFT.” (Participant 16, female, Associate Nurse Unit Manager, Medical ward).* Those who were not concerned also thought that staff had a good awareness of their own abilities, and that staff were very vocal and would speak up if they were not coping. For example, *“Staff are aware of their own abilities and a really good at coming forward and saying that they can’t cope.” (Participant 4, female, Clinical nurse specialist, special care nursery).* And also thought that staff were proactive and would just take sick leave if they were fatigued. For example, *“Staff are proactive and will take a sick day if needed.” (Participant 22, female, Associate Nurse Unit Manager, Speciality ward).* Or *“Not so worried about fatigue as staff are good at taking a sick day if they are tired or don’t want to do a shift. But are concerned about staff burnout.” (Participant 23, female, Nurse unit manager, Surgery ward).* However, some participants admitted that certain staff had a better understanding than others when identifying the signs of fatigue and acting upon these warning signs, stating, *“Some don’t want to admit they are not coping or don’t want to let the team down…. Some keep taking on more and more, so you have to tell them to stop. Staff take on extra shifts and burnout and don’t know it until it is too late, and they get rundown and sick!” (Participant 19, female, Nurse Unit Manager, Palliative care ward).* Whilst there were concerns, in the end if staff requested the shifts, then they were often granted, as one stated *“I am concerned about fatigue but if that is what they requested then so be it.” (Participant 17, female, Associate Nurse Unit Manager, Specialty ward).*

When it came to fatigue related workplace accidents, some participants who received the incident reports didn’t have fatigue as a standard reason that was considered when deciding what might be impacting on their health and wellbeing. For example, *“I receive all the reports that come in. I don’t ask if it was fatigue related.” (Participant 8, female, Associate Nurse Unit Manager, Rehabilitation unit).*

And that rosters do not change if there was an incident *“Schedules usually don’t change because of safety.” (Participant 17, female, Associate Nurse Unit Manager, Surgical ward).* Others were aware that the reports had the option for staff to pick fatigue as a contributing factor to an incident, but it was up to the staff member to flag whether they felt fatigue was an influence, for example, *“There is an option when reporting that staff can choose fatigue as a contributing factor.” (Participant 10, female, Nurse unit manager, Speciality ward).*

Additionally, some participants said that any workplace incident or medical error was seen as more of a competency and training matter rather than a fatigue-related issue, stating, *“…the reports should not go to the rostering staff because of privacy reasons. If there are more incidents, then it is a performance related issue not rostering. “(Participant 23 female, Nurse unit manager, Surgery ward)).* Additionally, some participants thought it was a private and confidential matter, therefore they should not be informed if a fatigue related incident was flagged by staff on their ward, such as *“I don’t think roster staff should get information due to privacy.” (Participant 20, female, Registered Nurse, ICU).*

### Fatigue responsibility

Many participants believed that because staff request the shifts, they should then also be responsible for the fatigue or safety issues that might arise from selecting particular shift patterns. For many participants there were too many staff members to be able to be across everyone and monitor their level of coping with the shift pattern allocated to them. As mentioned by one participant, *“Staff should be able to recognise their own fatigue because I can’t be across all the 50 + staff and how they are coping.” (Participant 22, female, Associate Nurse Unit Manager, Speciality ward).*Thus, most participants thought that staff should be responsible and that they were able to identify signs of fatigue, knew their limitations and would act upon them if needed. For example, *“Staff wouldn’t put themselves on a shift if they couldn’t cope.” (Participant 10*,*female, Nurse unit manager, Speciality ward)* or that *“Staff are not shy, are vocal if they are not coping.” (Participant 23, female, Nurse unit manager, Surgery ward).* However, there was acceptance by most participants that maybe some staff had a better understanding than others, with some mentioning that *“Some don’t want to admit it sometimes. Some staff will just soldier on even though they are tired and exhausted.” (Participant 19, female, Nurse Unit Manager, Palliative Care Unit).*

### Improvements or barriers to change

#### More flexibility/autonomy

When asked how rostering could be improved, participants wanted more flexibility and changes around what they could roster, with rosters having to be completed too far in advance for staff to plan that far out, resulting in more swapping and shift changes. *“6–8 weeks is too far out to think and request days off. This results in even more double-handling and shift changes”. (Participant 24, female, Associate Nurse Unit Manager, Speciality ward).*

Others wanted changes to software programs, so they didn’t have to double-handle tasks such transferring excel spreadsheet data into Kronos. *“[I] have to double-handle everything- excel to Kronos. Kronos is terrible, it doesn’t do what staff need it to do. For example, doesn’t tell you where the short falls are in each shift. New software updates at least will have the capacity to flag short falls in shifts to be able to put more staff on.” (Participant 23, female, Nurse unit manager, Surgery ward).*

#### Cultural barriers

Participants who sought to implement safer rostering practises, noted that they had received resistance when they have tried to implement changes. Staff attitudes and culture were the biggest barriers to change. For example, staff don’t want to give up some of the perks of shift work, including always finishing on an early shift so they felt like they had an extra day off or not waste their days off by being fatigued and discussed by a couple of participants saying *“It is hard to minimise late/early shifts when people preference to start on lates and end on earlies. We are trying to start to change this so that staff finish on late but not going well. They just swap anyway.” (Participant 22, female, Associate Nurse Unit Manager, Speciality ward).* or *“If staff are on-call, they want to work again the next day because staff don’t want a day off when they are fatigued as they don’t want to waste a day off.” (Participant 5, female, Community nurse, Community services department).*

There was also interest in knowing how other wards and hospitals are doing their rosters and the opportunity to learn from each other and was considered a positive solution to improving processes and outcomes, with one participant mentioning that *“Resilience training as staff can get angry about public holidays, weekends, and preferences etc. Also, would be good to share ideas of what others are doing with the rosters in different wards/hospitals. What strategies do they use? What have they tried?” (Participant 19, female, Nurse Unit Manager, Palliative Care Unit).*

## Discussion

To our knowledge this is the first study to document and report on the current rostering process of nurses in hospitals, how these staff are assigned and trained as well as the fatigue concerns and cultural/attitude barriers faced in changing processes. The findings from this study show that nurse rostering is complex and challenging. Staff who are responsible for doing the rosters need to be provided with more information and guidance on how to develop and implement safe shift work schedules to meet the legal obligations stated in the OH&S Act 2004 and 2014 [12], to minimize fatigue and workplace incidents. However, this is difficult to implement when staff are able to self-roster and dictate their shift work schedules. Consequently, there is an obligation for both the organisation and the individual staff requesting their shift schedules to understand fatigue risk and be accountable to contribute to a safe working environment.

### Preparation/education

The findings indicate that there is an absence of training or direction given to rostering staff by the organisations on how to create a safe shift work roster. All participants indicated that an internal hand-over from a previous staff member is commonly provided and staff learnt on the job about how to do the rostering. The OH&S Act clearly states that all levels of management are responsible to ensure that staff are trained on any specific knowledge and skills a person needs to fulfil their role effectively, or to manage new and/or temporary responsibilities [12]. The guidelines from the WorkSafe Victoria state that necessary training and information should include “body clock and sleep processes (including sleep hygiene and sleep disorders), risk factors for each type of fatigue (physical, mental and emotional), signs and symptoms of each type of fatigue in self and others, self-assessment tools and risk management strategies, procedures for preventing fatigue, such as incident reporting, health and lifestyle factors that may contribute to fatigue or impede good quality sleep, and balancing work and life demands” [10]. This study highlights that there is scope to improve the knowledge of rostering staff to improve the risk fatigue of staff.

### Consequences of fatigue

There is a strong reliance on staff to have the skills and knowledge to be able to identify their own levels of fatigue and to respond accordingly. In this study, participants responsible for rostering believed that staff had a good awareness of their own fatigue levels and self-care, with many stating that staff are very vocal and would speak up if they were not coping. However, some did admit that this does require confidence and the ability to recognise signs of fatigue. Individuals do not necessarily have the knowledge of how to manage the impact of shift work on their sleep, nor know what a safe shift work roster looks like. Increasing sleep education of healthcare workers has been shown to significantly improve sleep and fatigue [25–27]. Therefore, staff may not be considering the detrimental effects that inadequate shift schedules can have, not just on their health and wellbeing but also the safety of patients and instead preferencing for convenience or to fit in with other social and family commitments. This is highlighted by research that showed there was high work-family conflict especially among night shift workers and those working in a shift schedule [28].

Relying on staff to take mental health or sick leave days to cope with shift work is not a proactive strategy and impacts on a workers mental and physical health as well as the organization’s finances and the ability to deliver quality and safe care to patients. The annual cost of productivity loss to an organisation in Australia attributable to inadequate sleep is around $17.9 billion per year [3, 29]. Additionally, low retention rates are an issue in the healthcare industry, with significantly higher levels of fatigue and burnout amongst healthcare shift workers, with almost 70 per cent of Australian nurses struggling with fatigue and burnout in 2022 [30]. Nurses who also experience severe sleep disruption are more likely to leave their job within two years [31, 32]. With the healthcare system in a prolonged state of crisis, exacerbated by workforce shortages, poor retention adds additional costs to the organization, with the cost of advertising, onboarding, and training new employees needing to be considered [33, 34]. Additionally, fatigue and sleep impairment increase the risk of workplace accidents and medical errors, decreasing the safety of the patients [8, 35], with healthcare workers being less likely to acknowledge that fatigue is affecting their performance [36]. More needs to be done to ensure that healthcare shift workers are fit and healthy to ensure career longevity.

### Shift work patterns

Research affirms certain shift patterns are more detrimental to sleep and recovery than others [15, 35]. A clockwise shift rotation pattern (early, late, then nights) is highly recommended to minimize circadian disruption from shift work schedules [15, 37]. Also, night shifts should be no more than 3–4 consecutive nights with 2 days break in-between [15, 37]. However, safe shift work patterns seem to be ranked below other priorities such as staff preferences and entitlements. This is illustrated in this study where participants said that they wanted to change rosters but there was a culture where staff preferred to finish on an early shift because then they have the rest of the day free. Or same staff preferred to work the day after a night shift, so as to not waste their day off with feeling fatigued. Such attitudes highlight the disconnect between staff preferences and what shift patterns are best to optimise the delivery of safe, high-quality care. WorkSafe Victoria specifically outline criteria for safe shift work patterns to minimize fatigue risk including number of consecutive night shifts, minimum break in-between shifts and forward shift rotation [10], however, nurse and industry representatives and the enterprise agreements, which many participants quoted in this study, do not cover this in detail. For instance, they broadly state that, as far as practicable, employers must have regard for fatigue considerations and that employers and employees need to cooperate to develop safe rostering practices and prevention fatigue risk, however, how this is achieved and how this related to the priority of staff preferences was unclear. The ANMF do have guidelines on rostering practices [38] that state evidence-based research should be considered when rostering including minimum break hours between rostered shifts, shift rotations, the number of consecutive shifts of any type, but what these specifically are is unclear in the guidelines. This study found that, in practice, rostering staff are not aware of what evidence-based research is specifically to be able to create safe rosters. A clearer, stronger stance is needed by nursing organizations and in the EBA to precisely outline best practice guidelines by defining what shift rotation and shift work patterns are safest/ideal and to prioritize safety. Overall, a balance is needed which accommodates nurses’ personal circumstances and shift preferences to ensure a healthy work/life balance as well as ensuring the best interests of the organization and safety of the patients.

### Responsibility

While there is a responsibility for those creating the rosters to create safe shift work schedules, this can be challenging, especially when they have to roster for 50–100 + staff. It is an accepted, unspoken practice for a level of responsibility to reside with individual staff. The most common rostering practice was a self-rostering style process, which is perceived to be a fairer and more transparent way of rostering, empowering staff, and allowing them to be proactive in their work/life balance. Self-rostering was viewed by participants as a beneficial system that reduces stress and improves work/life balance because staff are able to choose shifts that fit best with their personal, social, and family situations. It is also seen as a safer way of creating rosters as staff can potentially determine the work patterns, they can best cope with. However, the risk is that staff might be more focused on how they can fit their shift work schedules into their lifestyle, disregarding how the shift schedules patterns may impact their fatigue and recovery. In spite of this, staff have been given the ability to request shifts, and are more likely to swap or just call in sick if not granted. Even though the OH&S Act 2004 and 2014 states that the responsibility lays with employers and management to provide information and training to mitigate fatigue risk, nursing staff also need to ensure they are fit for work. The Australian Health Practitioner Regulation Agency (AHPRA) states that registered nurses are also responsible in maintaining their physical and mental health to practise safely and effectively by acting to reduce the effect of fatigue and stress on their health, and on their ability to provide safe care [39]. Therefore, there needs to be a modification in the amount of influence staff have on their rosters and more focus put back on developing safe shift work schedules. The organisation needs to have policies and procedures in place to support and guide to ensure safe rostering are being created and train those responsible for the rosters on who to achieve this. If staff are to continue to contribute to their own rosters, they need to be educated on what is best practice and made aware of the consequences if they do not prioritise their health through minimising the likelihood of fatigue.

### Recommendations

Ultimately, rostering needs to be a two-pronged approach with employers and employees holding dual responsibility for creating safe shift work schedules. Organisations need to provide more guidance and training on rostering practices. The development of policies outlining ‘best practice’ for shift work scheduling would help bring the organisations in-line with legislative obligations. Employer driven sleep and shift work scheduling education has also been shown to reduce fatigue, sleep quality and burnout, and improve safety in shift workers [40, 41]. However, training of rostering staff will not necessarily reduce the fatigue risk. If staff have the autonomy to request and self-roster, they too must have an acceptable minimum level of knowledge and education, so that they understand the consequences and risks associated with requesting certain shift patterns.

Universities could also be doing more in preparing students for a career as a healthcare shift worker. Sleep is rarely covered in undergraduate nursing curriculum including good sleep hygiene practices, evidence-based coping strategies and safe shift work rosters would increase the understanding of why rules around shift work schedules are in place [42]. This may help decrease the resistance to change and help staff make more informed choices when they request certain shifts while mitigating the risks of sleep impairment and increasing alertness at work. Additionally, the power implications of staff rostering for their peers need to be considered, as there is the potential for favouritism or inequality of how rosters are created. Moving the rostering responsibility up to management-level could help shift the focus away from what the staff want and more to what is safe. Rostering is not a task that staff actively volunteer to undertake, which may be part of the reason for not doing it well or prioritising making sure their peers are happy.

## Conclusions

This study highlights that there are gaps in guidance and training from organisations for those responsible for creating the shift work rosters of nurses. Furthermore, while self-rostering resulted in staff having more freedom and flexibility, they may not be preferencing shifts that align to minimise fatigue and increase safety in the workplace. Accountability lies with all staff who should be educated on the signs of fatigue and how to mitigate fatigue risk from shift work schedules in the workplace. Greater consideration of the impact of shift work related fatigue is required to ensure that the layers of clinical governance in healthcare organisations minimise the risk of occupation health and safety issues for employees delivering direct patient care.

## Data Availability

The data from the interviews used to support the findings of this study are available from the corresponding author upon request.

## References

[1] Barger LK, Cade BE, Ayas NT, Cronin JW, Rosner B, Speizer FE (2005). Extended work shifts and the risk of motor vehicle crashes among interns. N Engl J Med.

[2] Rajaratnam SM, Howard ME, Grunstein RR (2013). Sleep loss and circadian disruption in shift work: health burden and management. Med J Aust.

[3] Sleep Health Foundation (2011). Re-awakening Australia: the economic cost of sleep disorders in Australia.

[4] Liu Y, Croft JB, Wheaton AG, Perry GS, Chapman DP, Strine TW (2013). Association between perceived insufficient sleep, frequent mental distress, obesity and chronic diseases among US adults, 2009 behavioral risk factor surveillance system. BMC Public Health.

[5] Gander P, O’Keeffe K, Santos-Fernandez E, Huntington A, Walker L, Willis J (2019). Fatigue and nurses’ work patterns: an online questionnaire survey. Int J Nurs Stud.

[6] Kecklund G, Axelsson J. Health consequences of shift work and insufficient sleep. BMJ. 2016;355. 10.1136/bmj.i5210 27803010

[7] Banks S, Landon LB, Dorrian J, Waggoner LB, Centofanti SA, Roma PG (2019). Effects of fatigue on teams and their role in 24/7 operations. Sleep Med Rev.

[8] Di Muzio M, Dionisi S, Di Simone E, Cianfrocca C, Di Muzio F, Fabbian F (2019). Can nurses’ shift work jeopardize the patient safety? A systematic review. Eur Rev Med Pharmacol Sci.

[9] Uehli K, Mehta AJ, Miedinger D, Hug K, Schindler C, Holsboer-Trachsler E (2014). Sleep problems and work injuries: a systematic review and meta-analysis. Sleep Med Rev.

[10] Victoria W. A guide for employers- Work-related fatigue Geelong, Victoria: WorkSafe victoria; 2020 [ https://www.worksafe.vic.gov.au/work-related-fatigue-guide-employers-0.

[11] Safe Work Australia (2013). Guide for managing the risk of fatigue at work.

[12] Australia Po. Occupational Health and Safety Act 2004. editor. Canberra, Australia: Victorian Government; 2004. Parliment of Victoria.

[13] Occupational Health and Safety Amendment Regulations. 2017, S.R. No. 22/2017 (2017).

[14] van Amelsvoort LG, Jansen NW, Swaen GM, van den Brandt PA, Kant I (2004). Direction of shift rotation among three-shift workers in relation to psychological health and work-family conflict. Scand J Work Environ Health.

[15] Wangsan K, Chaiear N, Sawanyawisuth K, Klainin-Yobas P, Simajareuk K, Boonsawat W. Which Shiftwork pattern is the strongest predictor for poor sleep quality in nurses? Int J Environ Res Public Health. 2022;19(21). 10.3390/ijerph192113986 PMC9658910 36360864

[16] Viitasalo K, Kuosma E, Laitinen J, Härmä M (2008). Effects of shift rotation and the flexibility of a shift system on daytime alertness and cardiovascular risk factors. Scand J Work Environ Health.

[17] Sallinen M, Kecklund G (2010). Shift work, sleep, and sleepiness — differences between shift schedules and systems. Scand J Work Environ Health.

[18] Stake RE. The art of case study research: sage; 1995.

[19] Crowe S, Cresswell K, Robertson A, Huby G, Avery A, Sheikh A (2011). The case study approach. BMC Med Res Methodol.

[20] Yin RK. Case study research: design and methods. sage; 2009.

[21] Chapman AL, Hadfield M, Chapman CJ (2015). Qualitative research in healthcare: an introduction to grounded theory using thematic analysis. J R Coll Physicians Edinb.

[22] Birks M, Mills J. Grounded theory: a practical guide. Sage; 2015.

[23] Nowell LS, Norris JM, White DE, Moules NJ (2017). Thematic analysis: striving to meet the trustworthiness criteria. Int J Qualitative Methods.

[24] Stake RE. Multiple case study analysis. Guilford Press; 2013.

[25] Booker LA, Sletten TL, Barnes M, Alvaro P, Collins A, Chai-Coetzer CL (2022). The effectiveness of an individualized sleep and shift work education and coaching program to manage shift work disorder in nurses: a randomized controlled trial. J Clin Sleep Med.

[26] Mazar D, Gileles-Hillel A, Reiter J (2021). Sleep education improves knowledge but not sleep quality among medical students. J Clin Sleep Med.

[27] Burton WN, Chen C-Y, Li X, McCluskey M, Erickson D, Barone D (2016). Evaluation of a workplace-based Sleep Education Program. J Occup Environ Med.

[28] Wöhrmann A, Müller G, Ewert K. Shift Work and Work-Family Conflict: A Systematic Review. sozialpolitikch. 2020;2020.

[29] Sleep Health Foundation (2017). Asleep on the job: costs of inadequate sleep in Australia.

[30] Australian College of Nursing (2022). Nurse leadership during disruptive events – A report by the Australian College of Nursing and Health Professionals Bank 2022.

[31] Han K, Kim Y-H, Lee HY, Lim S (2020). Novice nurses’ sleep disturbance trajectories within the first 2 years of work and actual turnover: a prospective longitudinal study. Int J Nurs Stud.

[32] Hayes LJ, O’Brien-Pallas L, Duffield C, Shamian J, Buchan J, Hughes F (2012). Nurse turnover: a literature review - an update. Int J Nurs Stud.

[33] Roche MA, Duffield CM, Homer C, Buchan J, Dimitrelis S (2015). The rate and cost of nurse turnover in Australia. Collegian.

[34] Mills J, Chamberlain-Salaun J, Harrison H, Yates K, O’Shea A (2016). Retaining early career registered nurses: a case study. BMC Nurs.

[35] Ganesan S, Magee M, Stone JE, Mulhall MD, Collins A, Howard ME (2019). The impact of Shift Work on Sleep, alertness and performance in Healthcare Workers. Sci Rep.

[36] Sexton JB, Thomas EJ, Helmreich RL (2000). Error, stress, and teamwork in medicine and aviation: cross sectional surveys. BMJ.

[37] Berger AM, Hobbs BB (2006). Impact of Shift Work on the Health and Safety of nurses and patients. Clin J Oncol Nurs.

[38] Australian Nursing and Midwifery Federation (Victorian Branch). Rostering2019. https://anmf.org.au/documents/policies/P_Rostering.pdf.

[39] Nursing and Midwifery Board of Australia (NMBA). Safety and quality guidelines for nurse practitioners. editor. Canberra, Australia: Nursing and Midwifery Board of Australia (NMBA),; 2021. Australian Health Practitioner Regulation Agency (AHPRA).

[40] Redeker NS, Caruso CC, Hashmi SD, Mullington JM, Grandner M, Morgenthaler TI (2019). Workplace interventions to promote sleep health and an alert, healthy workforce. J Clin Sleep Med.

[41] Barger LK, Runyon MS, Renn ML, Moore CG, Weiss PM, Condle JP (2018). Effect of fatigue training on safety, fatigue, and Sleep in Emergency Medical Services Personnel and other Shift workers: a systematic review and Meta-analysis. Prehospital Emerg Care.

[42] House of Representatives Standing Committee on Health ACaS. Bedtime Reading: Inquiry into Sleep Health Awareness in Australia Canberra, Australia: Parliment of the Commonwealth of Australia. 2019 [ https://www.sleephealthfoundation.org.au/files/Parliamentary_Inquiry/BedtimeReading-Parliamentary_Report.pdf.

